# Supplementary material for: The interactome of intact mitochondria by cross-linking mass spectrometry provides evidence for coexisting respiratory supercomplexes
Source: Mol Cell Proteomics. 2017 Dec 8;17(2):216–32. doi: 10.1074/mcp.RA117.000470 (PMC5795388; doi:10.1074/mcp.RA117.000470)
Supplement: Supplemental Data [file supp_RA117.000470_133922_0_supp_23978_fzffwf.zip › spectra_annotation/mito_spectra_annotation/212-1-2-1-8-1.pdf]

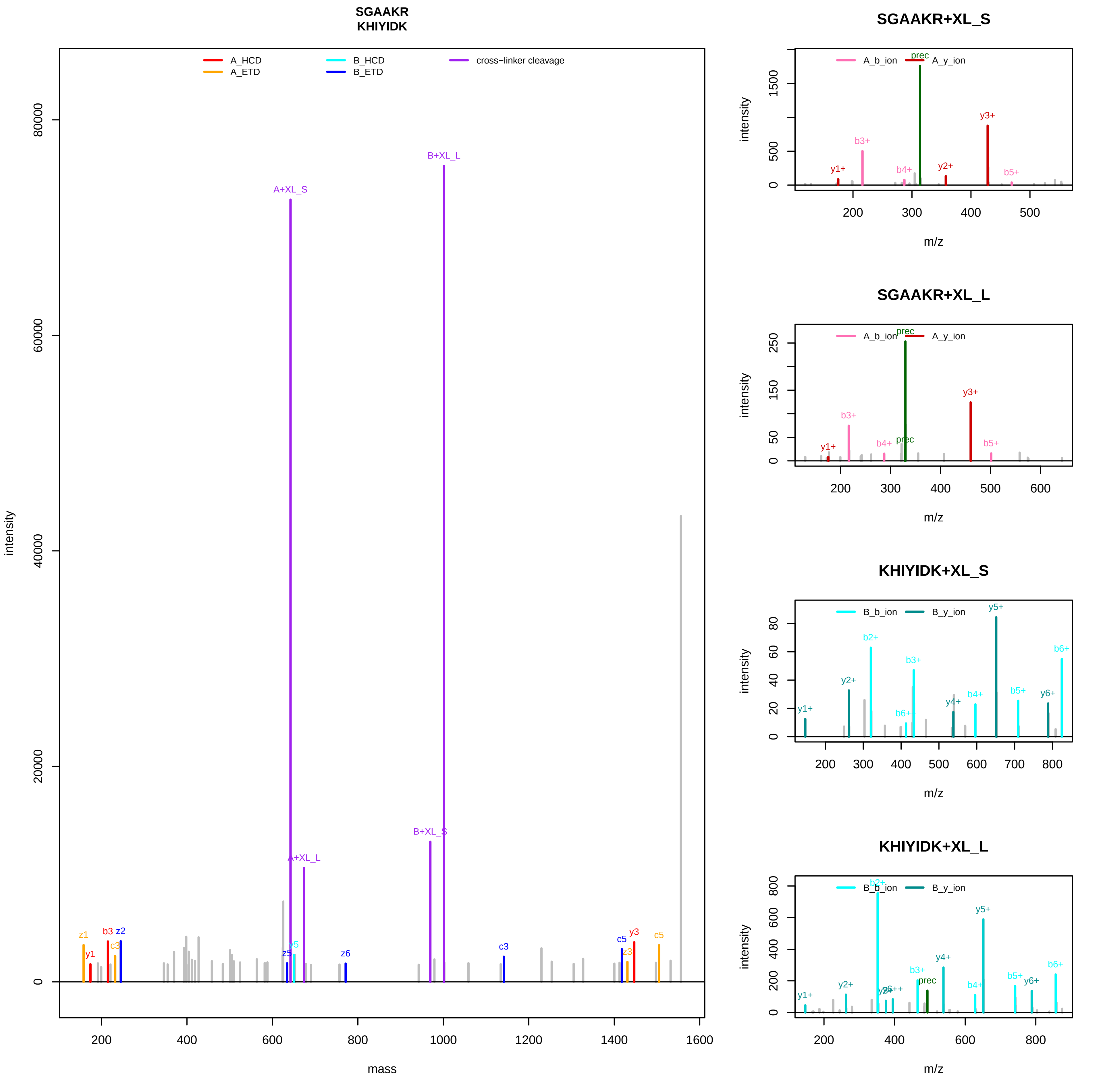
SGAAKR
KHIYIDK
A_HCD
A_ETD
B_HCD
B_ETD
cross−linker cleavage
intensity
mass
0
20000
40000
60000
80000
200
400
600
800
1000
1200
1400
1600
A+XL_S
B+XL_L
B+XL_S
A+XL_L
z1
y1
b3
c3
z2
z5
y5
z6
c3
c5
z3
y3
c5
SGAAKR+XL_S
A_b_ion
A_y_ion
prec
y1+
b3+
b4+
y2+
y3+
b5+
m/z
SGAAKR+XL_L
KHIYIDK+XL_S
B_b_ion
B_y_ion
y5+
b2+
b3+
y2+
b6+
y1+
y4+
b4+
b5+
y6+
KHIYIDK+XL_L
